# Light at the ENDothelium-role of Sox17 and Runx1 in endothelial dysfunction and pulmonary arterial hypertension

**DOI:** 10.3389/fcvm.2023.1274033

**Published:** 2023-11-02

**Authors:** Robert Simmons Beck, Olin D. Liang, James R. Klinger

**Affiliations:** ^1^ Division of Pulmonary, Sleep and Critical Care Medicine, Rhode Island Hospital and the Alpert Medical School of Brown University, Providence, RI, United States; ^2^ Division of Hematology/Oncology, Rhode Island Hospital and the Alpert Medical School of Brown University, Providence, RI, United States

**Keywords:** pulmonary hypertension, endothelial dysfunction, transcription factors, SOX17, Runx1, pulmonary vascular remodeling, vasculogenesis

## Abstract

Pulmonary arterial hypertension (PAH) is a progressive disease that is characterized by an obliterative vasculopathy of the distal pulmonary circulation. Despite significant progress in our understanding of the pathophysiology, currently approved medical therapies for PAH act primarily as pulmonary vasodilators and fail to address the underlying processes that lead to the development and progression of the disease. Endothelial dysregulation in response to stress, injury or physiologic stimuli followed by perivascular infiltration of immune cells plays a prominent role in the pulmonary vascular remodeling of PAH. Over the last few decades, our understanding of endothelial cell dysregulation has evolved and brought to light a number of transcription factors that play important roles in vascular homeostasis and angiogenesis. In this review, we examine two such factors, SOX17 and one of its downstream targets, RUNX1 and the emerging data that implicate their roles in the pathogenesis of PAH. We review their discovery and discuss their function in angiogenesis and lung vascular development including their roles in endothelial to hematopoietic transition (EHT) and their ability to drive progenitor stem cells toward an endothelial or myeloid fate. We also summarize the data from studies that link mutations in Sox17 with an increased risk of developing PAH and studies that implicate Sox17 and Runx1 in the pathogenesis of PAH. Finally, we review the results of recent studies from our lab demonstrating the efficacy of preventing and reversing pulmonary hypertension in animal models of PAH by deleting RUNX1 expression in endothelial or myeloid cells or by the use of RUNX1 inhibitors. By investigating PAH through the lens of SOX17 and RUNX1 we hope to shed light on the role of these transcription factors in vascular homeostasis and endothelial dysregulation, their contribution to pulmonary vascular remodeling in PAH, and their potential as novel therapeutic targets for treating this devastating disease.

## Introduction

The pulmonary circulation is a low-pressure circuit with a mean pulmonary arterial pressure (mPAP) of approximately 14 mmHg. Elevation of mPAP > 20 mmHg is defined as pulmonary hypertension and is common in patients with chronic heart and lung diseases (1, 2), occurring up to 50%–70% of patients. When pulmonary hypertension is accompanied by an elevation in pulmonary vascular resistance (PVR) and occurs in the absence of elevated left sided filling pressures and significant heart or lung disease, it is referred to as pulmonary arterial hypertension (PAH). This term is used to distinguish the elevated PA pressure as being caused by a disease of the pulmonary arterial circulation as opposed to a consequence of chronic hypoxia, parenchymal lung disease or elevated pulmonary venous pressure. PAH is a complex, heterogeneous, and frequently fatal disease that results from progressive functional and structural changes in the pulmonary vasculature that lead to increased PVR, right ventricular failure and usually death. Although response to treatment can vary considerably between patients, overall survival from diagnosis averages only about 5 years.

The disease is rarely seen in the general population with an annual incidence of less than 1.5 per 100,000 healthy individuals but occurs in 1%–10% of patients with connective tissue disease, portal hypertension, or HIV infection (3). It is also associated with congenital left to right intra-cardiac shunts, and in patients with a history of fenfluramine/phentermine or methamphetamine use. When seen in conjunction with one of these conditions, the disease is described as associated PAH (APAH). There are also a number of gene mutations that greatly increase the risk of developing PAH and patients with one of these mutations and a family history of PAH are described as having heritable PAH (HPAH). PAH patients without APAH or HPAH are described as having idiopathic PAH (IPAH). Despite a comprehensive knowledge regarding clinical characteristics of PAH, the cellular injury or stressors and mechanisms responsible for disease development are not well understood.

At the turn of century, heterozygous germline mutations in the gene encoding bone morphogenetic protein receptor type 2 (BMPR2) were found to be associated with familial PAH. Mutations in BMPR2 have since been recognized as occurring in approximately 70% of HPAH (4, 5) and 10%–20% of IPAH cases (6, 7). However approximately 75% of cases of PAH cannot be explained by BMPR2 mutations alone and only about 1 in 8 people carrying one of the disease variant BMPR2 mutations will develop the disease (8). Thus, it is believed that PAH may be the result of a double hit phenomenon consisting of an initial injury to the pulmonary circulation combined with an impaired reparative response due either to a gene mutation or other disease conditions that facilitate abnormal pulmonary vascular remodeling. Since the initial reports of BMPR2 mutations, whole exome and whole genome sequencing has led to the identification of more than 16 genes that have been associated with PAH. Many of these (e.g., Krüppel-like factor 5 (KLF2), GATA-binding factor 2 (GATA2), T-box 4 (TBX4), SRY-box 17 (Sox17), eukaryotic initiation translation factor 2 ɑlpha kinase 4 (EIF2AK4)) are transcription factors or transcriptional coactivators/repressors that play important roles in lung and/or vascular development or vascular homeostasis (9–12).

**Figure 1:**
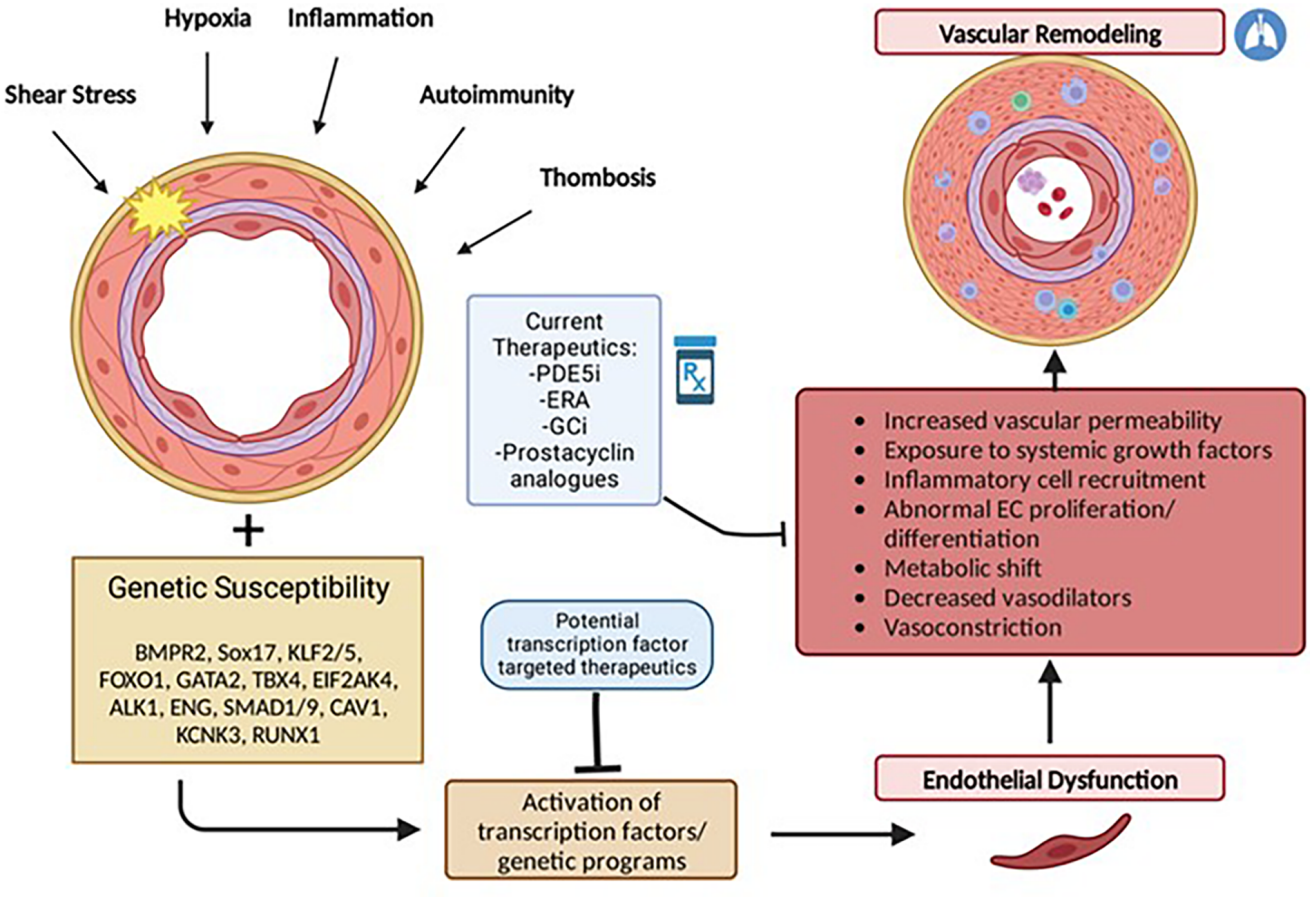
Multiple hit model of pulmonary arterial hypertension development. PAH results from endothelial dysfunction and subsequent vascular remodeling triggered by activation or suppression of transcription factors in response to repetitive stressful stimuli. Individual genetic susceptibility (i.e., genetic mutations, gender and epigenetic factors) predisposes endothelial cells to cellular dysregulation. Current therapeutics act primarily in response to vascular remodeling and as pulmonary vasodilators but largely do not reverse pulmonary vascular remodeling characteristic of PAH.

Although the current understanding of PAH pathophysiology remains limited, an emerging hypothesis is that vascular injury in response to a variety of factors such as hypoxia, shear stress, inflammation, oxidative stress, or growth factors, leads to abnormal proliferation or differentiation of pulmonary vascular cells including endothelial cells, smooth muscle cells, and adventitial fibroblasts. Under normal conditions, cellular stress or injury may trigger downstream activation of genetic programs involved in angiogenesis and neovascularization that are important for restoring vascular integrity. These programs are mediated by specific transcription factors which if not properly expressed may lead to abnormal pulmonary vascular remodeling (Figure 1).

Currently approved medical therapies for PAH, have some ability to inhibit cellular proliferation and hypertrophy, but act primarily as pulmonary vasodilators and have limited ability to repress the pathogenic phenotypes of pulmonary vascular cells. Given their vital roles in angiogenesis and vascular homeostasis, several transcription factors have emerged as enticing therapeutic targets to potentially counteract and reverse the PAH vascular phenotype.

In this review we focus on two transcription factors with emerging roles in PAH: SOX17 and one of its downstream targets, RUNX1. We examine their normal structure and functions, and their roles in EHT, endothelial progenitor cell differentiation, and genetic mutations associated with PAH. By examining the role of SOX17 and RUNX1 in the pathogenesis of PAH we hope to further our understanding of the cellular mechanisms that drive remodeling of damaged lung vessels in pulmonary vascular disease and propose potential future therapeutic targets for the treatment of patients with PAH.

## SOX17

In 1990, the SRY gene—sex-determining region of the Y-chromosome was first discovered in humans and mice as a testis-determining gene (13, 14). The identification of a highly conserved high-mobility group (HMG) domain led to the discovery of the SRY-box (SOX) transcription factor family. Over the following decades, gain and loss of function studies in the 20 members of the Sox transcription factor family revealed that they played a crucial role in the regulation of various developmental processes, organogenesis, cell fate determination and tissue homeostasis (15, 16).

**Figure 2:**
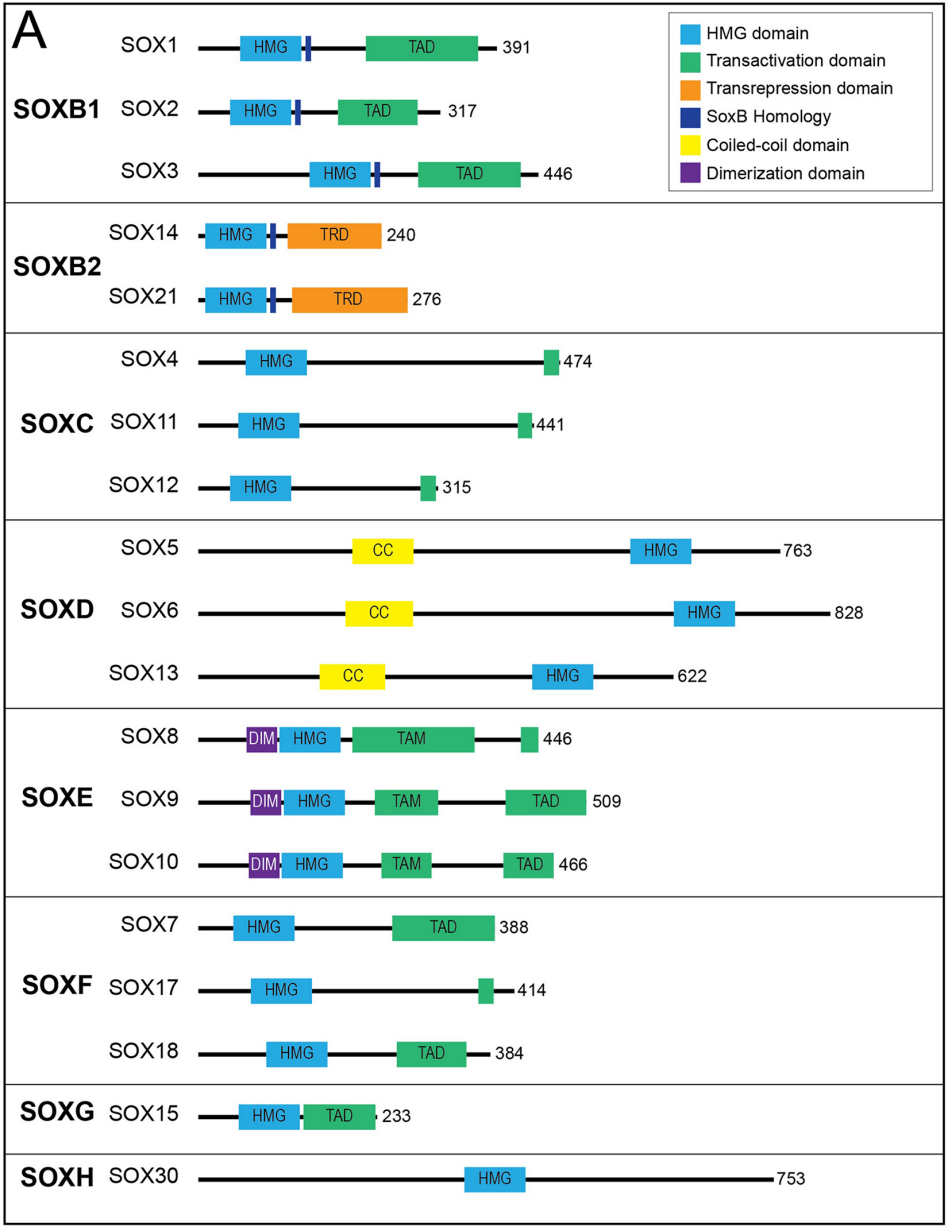
Sox transcription factor subfamilies and their functional domains. SOX proteins grouped by subfamily. Major protein functional domains are depicted as colored boxes: High-mobility group (HMG) domain (light blue), transactivation domain (TAD; green), transrepression domain (TRD; orange), SoxB homology domain (dark blue), coiled-coil (CC) domain (yellow), and dimerization (DIM) domain (purple). Reproduced from Schock EN, LaBonne C. Sorting Sox: Diverse Roles for Sox Transcription Factors During Neural Crest and Craniofacial Development. Front Physiol. (2020 Dec 8) 11:606889. doi: 10.3389/fphys.2020.606889. PMID: 33424631; PMCID: PMC7793875.

The high-mobility group (HMG) domain in SOX genes allows for DNA binding in a sequence-specific manner. Each of the SOX family of genes encodes an HMG domain of 79 amino acids that is highly conserved throughout eukaryotic species and enables their binding to a specific DNA sequence (A/T A/T CAA A/T) (17). SOX genes are classified into groups A-H based on phylogenetic differences in their HMG box sequences, protein structure and involvement in developmental processes (Figure 2) (18). Within SOX subfamilies, the structural domains of the proteins outside the HMG domain are similar but not identical. These domains include the transactivation domain (TAD), the transrepression domain (TRD), coiled-coil domain (CC) and the dimerization domain (DIM). The TAD or TRD are domains that contain binding sites for other proteins (i.e., transcription coregulators) to bind, whereas DIM and CC allow for dimerization of Sox proteins (19). In contrast to most transcription factors, Sox proteins introduce strong bends into DNA, thereby allowing multiple regulatory proteins and transcription factors to bind by colocalization with enhancers or gene promoters (20). Importantly, many Sox family proteins are pioneer factors, a subset of transcription factors that serve as master regulators that can bind to target DNA sequences even when they are buried inside condensed chromatin and can thereafter initiate opening of closed chromatin and activation of transcription (21). Interestingly, SOX genes appear to play critical roles in the generation and induction of pluripotent stem cells (PSCs) and embryonic stem cells (ESCs)—regulating pluripotency and mediating self-renewal and differentiation to endothelial cells (21, 22).

SRY-box transcription factor 17 (SOX17) is a member of the SOXF subfamily along with SOX7 and SOX18. All members of this subfamily contain a TAD in the C-terminal and a short functional motif (DxxEFD/EQYL) thought to be involved in the interaction with Beta-catenin (18). Members of this group have been shown to be crucial in early cardiovascular and hematopoietic cell development in mice and humans and are heavily expressed in vascular endothelial precursors and progenitor cells (see section below on endothelial progenitor cells). All members of the SOXF subgroup are expressed in endothelial cells during development and frequently have overlapping roles in the development of the cardiovascular system (21).

The human *SOX17* gene contains two exons expressed in human heart, lung, spleen, testis, ovary and placental and in fetal lung, and kidney tissue. Although widely expressed during development, *SOX17* displays a largely endothelial-specific expression profile in healthy adult tissues (23). In mouse embryonic tissue, *Sox17* expression is initially localized to the endoderm but subsequently increases in the dorsal aorta during vascular development. In adult mice, expression is largely contained to arterial vascular endothelium rather than venous endothelium (24).

**Figure 3:**
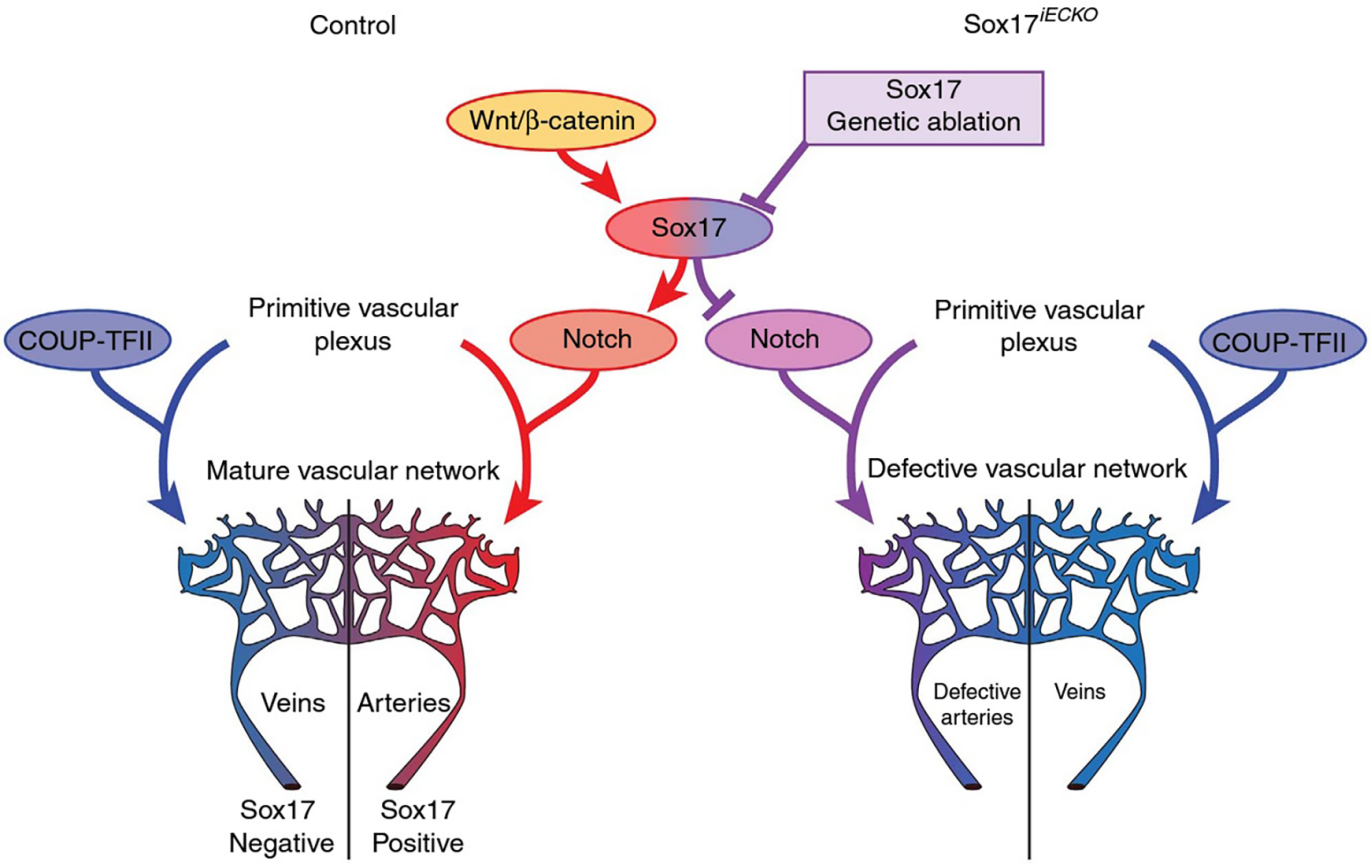
Representation of potential signaling pathways regulating arterial/venous specification. Wnt/Beta-catenin signaling activates Notch signaling and arterial differentiation through Sox17. Endothelial-specific inactivation of Sox17 prevents Notch signaling and acquisition of arterial identity by endothelial cells. COUP transcription factor II (COUP-TFII—important for venous determination), Sox17 iECKO (Sox17 endothelial cell specific knock out at P1) Wnt. Reproduced from Corada et al., 2016 2609.

SOX17 is a crucial endothelial-specific transcription factor involved in arteriovenous differentiation, pulmonary vascular morphogenesis, angiogenesis, and pulmonary endothelial regeneration following vascular injury. *Sox17* and *Sox18* both appear to be necessary for vascular endothelial postnatal angiogenesis, whereas low *Sox17* and high *Sox7*/*Sox18* expression are necessary for systemic vein development (25). *Sox17* is necessary for arterial differentiation and vascular development and promotion of arterial identity appears to be mediated via downstream activation of the Notch pathway (Figure 3). Endothelial cell-specific deletion of *Sox17* in mice leads to death *in utero* associated with lack of large artery formation, formation of defective vascular networks including arterio-venous malformations, and lack of arterial and venous differentiation (21). Cell-specific knockout of SOX17 in developing mouse pulmonary vascular endothelium causes the development of pulmonary vein varices, dilated pulmonary arteries and abnormal lung perfusion along with biventricular hypertrophy (23).

SOX17 expression remains high in arteries of mature mice, supporting a role for not only induction of artery formation but also maintenance of mature vasculature identity. Interestingly, single nucleotide polymorphisms in the *SOX17* gene have been associated with increased risk of intracranial aneurysm development in humans (26). In animal models, endothelial-specific SOX17 loss of function has been shown to result in impaired endothelial integrity, proliferation, paracrine regulation and increased development of intracranial aneurysms in response to hypertensive stress (27). Loss of Sox17 function appears to disrupt cell to cell adhesion via decreased VE-cadherin expression—a possible explanation for both intracranial aneurysm development and non-functional vascular networks with decreased Sox17 function (22).

*Sox17* also appears to actively prevent the transition from hematopoietic fate by repression of key hematopoietic transcription factors, thereby allowing cells to maintain arterial identity (See section below on endothelial to hematopoietic transition). Expression of SOX17 appears to be crucial in maintenance of endothelial rather than hematopoietic cell fate, as studies have demonstrated that tissue-specific, temporally controlled, knockout of arterial genes (SOX17 and NOTCH1) causes an increase in hematopoietic cells due to loss of Sox17 dependent repression of hematopoietic transcription factors Runx1 and Gata2 in both human and mice stem cells (28). *Sox17* is also crucial for maintaining a population of intra-aortic hematopoietic cluster (IAHCs) and fetal liver hematopoietic stem cells (HSCs) during development (28). Importantly, when SOX17 is downregulated during development in mouse embryonic days 9–11, hematopoietic cell differentiation is increased in cells in which *Sox17* is downregulated. So, although *Sox17* is crucial to the development of the endothelium, it also appears to be important in suppressing differentiation of embryonic endothelial cells towards a hematopoietic fate (28). Interestingly, while Sox17 binds directly to the *Runx1* and *Gata2* promotors to suppress their expression (28), Runx1 appears to repress Sox17 expression, indicating the presence of a negative feedback loop which appears to be important in directing endothelial progenitor cells toward an endothelial or hematopoietic fate (28).

## Role of *Sox17* in vascular homeostasis and neovascularization

While *Sox17* is responsible for endothelial development, differentiation and identity, it also plays a critical role in endothelial regeneration and homeostasis in response to injury. Activation of developmental pathways is key to tissue regeneration in response to tissue injury. Accordingly, Sox17 has been shown to be upregulated following inflammatory induced vascular injury and is necessary for endothelial regeneration thereafter. For example, SOX17 expression was diminished in intracranial aneurysms of adults undergoing microsurgical clipping, whereas it was highly expressed in intracranial arteries from controls (27). Similarly, *Sox17* has been shown to be necessary for vascular regeneration in response to vessel wall injury in adult mice (29). Activation of *Sox17* in response to inflammation induced vascular injury appears to be dependent on hypoxia inducible factor 1ɑ (HIF-1ɑ) signaling (29). *Sox17* overexpression in endothelial cells via liposomal cDNA delivery enhances endothelial cell and mouse survival in response to LPS-induced lung injury (29).

Sox17 may also promote the development of progenitor cell behavior in multiple adult cell types (30). Sox17 has been demonstrated to play a key role in maintenance of pluripotency and endothelial differentiation potential. It has been shown to be central in trans-differentiation of fibroblasts to endothelial cells via dedifferentiation into CD34 + progenitor cells in response to lung injury (29). Endothelial-specific deletions of Sox17 result in impaired endothelial cell junctional assembly, cell matrix adhesion and proliferative/regenerative capacity (28). *SOX17* overexpression has also been found to give rise to fetal-like HSCs with high self-renewal capacity (31). Similarly, *SOX17* appears to be crucial for maintenance of endothelial potential/differentiation in human pluripotent stem cells (hPSCs) (32). SOX17 has also been shown to upregulate tumor angiogenesis via increased VEGFR2 expression and inhibition of Sox17 leads to marked decreases in tumor progression, angiogenesis, and vascular density in multiple tumor models (33).

The signaling pathways by which SOX17 promotes progenitor cell and endothelial identities is not well understood. SOX17 appears to suppress SMAD3/TGF-β signaling—a pathway known as a potent inhibitor of epithelial cell proliferation. Interestingly, SOX17 mediated repression of hematopoietic lineage in EHT also appears to happen through TGF-β signaling (30). Recent studies have shown that SOX17 knockdown reduces BMPR2 expression and BMP9-induced phosphorylation of Smad1/5/9 (34) supporting a role for Sox17 in enhancing BMP signaling. Conversely, BMP signaling has been shown to repress Sox17 expression in zebrafish endoderm, whereas TGF-β signaling activates Sox17 signaling (35). Thus, BMP and Sox17 appear to form a feedback loop that modulates pluripotent stem cells differentiation. However, BMPs and SOX17 have significant overlap in their signaling effector targets in vascular cells, including VEGF, Notch, SMAD and Wnt signaling (36).

## Role of SOX17 in PAH

The healthy pulmonary vascular endothelium is designed to dynamically adapt to stressful stimuli such as shear stress, inflammation, or hypoxia. Under circumstances with repetitive injury and/or enhanced genetic susceptibility, the protective endothelial layer breaks down and can take on a proliferative, vasoconstrictive, and proinflammatory phenotype. This dysfunctional endothelium is thought to be an early trigger in PAH. Recent, genome-wide association data have demonstrated significant increased risk of PAH from genetic variants in *SOX17* enhancer regions (37). Similarly, prior data also demonstrated an association of deleterious mutations in *SOX17* with PAH (38, 39). Considering the endothelial cell-specific nature of SOX17, its role in neovascularization and maintenance of arterial identity, the increase risk of PAH in patients with loss of function mutations in *SOX17,* strongly suggest that this transcription factor plays a pivotal role in the pathogenesis of the disease.

**Figure 4:**
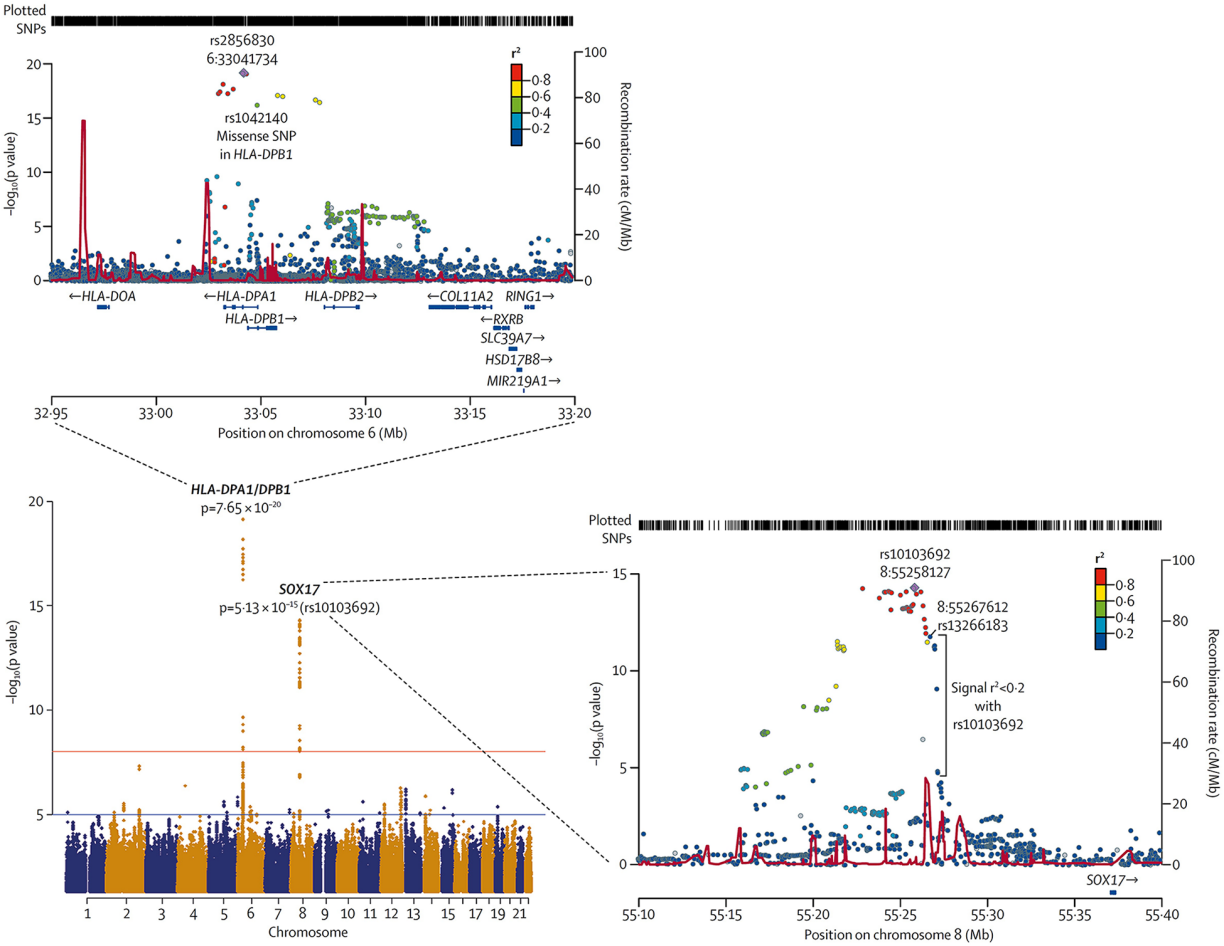
Meta-analysis of all cohorts (total 2,085 PAH patients and 11,744 controls) and regional plots of novel loci identified as being more frequently associated with PAH. Regional plots indicate variant location and linkage disequilibrium structure at SOX17 locus. Several variants associated with pulmonary arterial hypertension are in very weak or no linkage disequilibrium (r2 < 0·2) with the lead single-nucleotide polymorphism (SNP), rs10103692. Variants are referred to as SOX17 signal 1 and the most significant variant, rs13266183. The variants coloured as in linkage disequilibrium with rs10103692 comprise signal 2. Reproduced from Rhodes et al., 2019.

**Figure 5:**
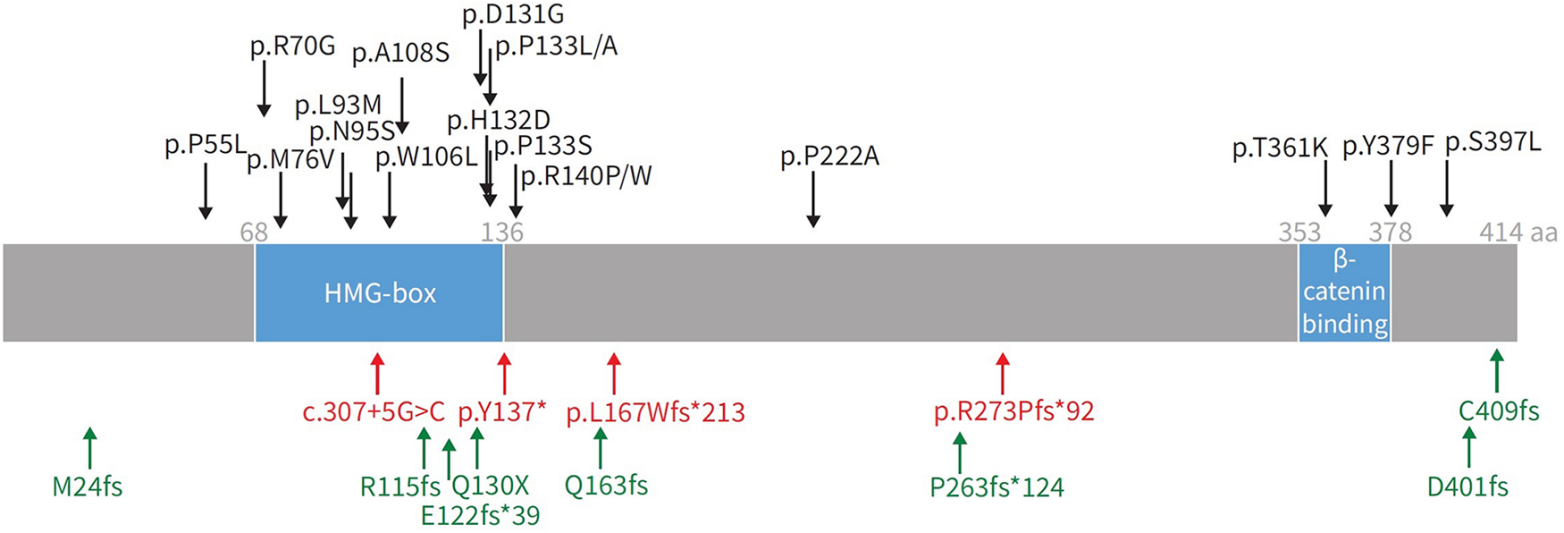
Representation of SOX17 gene with overlying variants identified in PAH patients. aa: amino acids; HMG: high mobility group. Black: missense mutations; red: protein-truncating variants; green: likely gene-disrupting variants. Reproduced from Wu, Y., Wharton, J., Walters, R., Vasilaki, E., Aman, J., Zhao, L., Wilkins, M. R., & Rhodes, C. J. (2021). The pathophysiological role of novel pulmonary arterial hypertension gene SOX17. The European respiratory journal. 58(3): 2004172. doi: 10.1183/13993003.04172-2020.

**Figure 6:**
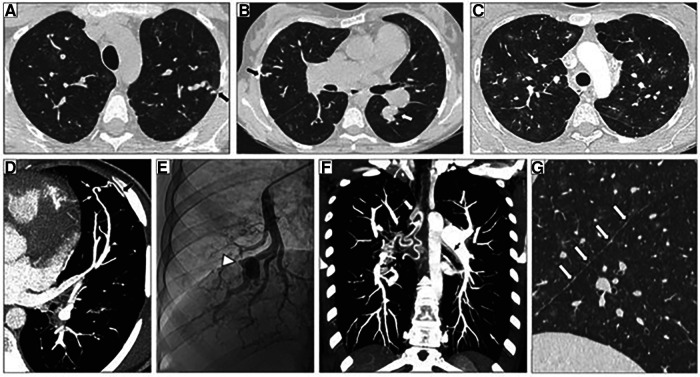
Representative high-resolution computed tomography (HRCT) of the chest, CT pulmonary angiography (CTPA) and pulmonary angiogram of pulmonary arterial hypertension patients carrying a SOX17 pathogenic variant. (**A–C**) Thin-collimated HRCT of the chest showing (**A,B**) subpleural dilated and tortuous pulmonary vessels (black arrows) and (**C**) ground-glass opacities. (**D**) The black arrow points to direct communication between a dilated distal pulmonary artery and a dilated intercostal artery. (**B,E**) Aneurysmal dilatation identified on b) CTPA (white arrow) and (**E**) pulmonary angiography (arrowhead). (**F**) Marked dilatation of proximal bronchial arteries is frequently observed (white and black arrows). (**G**) The presence of numerous fissural irregularities (white arrows) suggests the additional presence of dilated systemic vessels at the pleural surface. Reproduced from Montani et al., 2022.

In 2018, Graf et al., first identified *SOX17* mutations as being significantly over-represented in whole-genome sequencing of 1,038 PAH patients compared to 6,385 control subjects (38). Missense mutations and protein truncating variants in *SOX17* were heavily over-represented in PAH patients (Figure 4). Interestingly, patients with *SOX17* mutations were significantly younger at the time of diagnosis compared to other PAH patients. Mutations identified in *SOX17* gene were predicted to lead to loss of function of the beta-catenin region or suppress beta-catenin or Oct4 binding (38). Furthermore, SOX17 expression was isolated largely to the pulmonary endothelium in the lungs of healthy people and to the endothelial cells within plexiform lesions in patients with PAH. Shortly after Graf et al.'s work, Zhu et al., demonstrated an enrichment of rare deleterious *SOX17* mutations in patients with PAH associated with congenital heart disease (PAH-CHD) (3.2% of patients) and in patients with IPAH/HPAH (0.7% of patients). Interestingly, the majority of the missense mutations occurred in the highly conserved HMG-box protein domain responsible for DNA binding (39). Additionally, they also demonstrated enrichment of missense mutations in downstream genes targeted by SOX17 in PAH patients (39). More recently, a genome-wide association study of 11,744 European individuals (2,085 patients) identified 2 independent risk variant-containing signals (Sox17-signal 1 and Sox17-signal 2) in an enhancer region (Figure 5) near *SOX17* that were associated with PAH despite the relatively rare prevalence of mutations in the coding region of the *SOX17* gene itself—implicating a more common causative role for Sox17 in PAH development than previous thought (39). In that study, 59% of patients with PAH were homozygous for both risk alleles compared to 47% of controls. Furthermore, PAH patients with *SOX17* mutations demonstrate high rates of dilated and tortuous pulmonary vessels, hemoptysis and atrial and ventricular septal defects (Figure 6). These patients frequently present with severely compromised hemodynamic parameters at the time of diagnosis with a mean mPAP of 67 mmHg and PVR of 14 WU in one European cohort (40).

## Potential pathogenic mechanisms for SOX17 in PAH

Since the discovery of *SOX17* mutations and their association with PAH, investigators have sought to understand the mechanism by which impaired *SOX17* expression predisposes patients to PAH. Animal models and in-vitro studies have replicated many of the pathophysiologic hallmarks observed in human PAH patients in Sox17 deficient mice (Table 1). Studies using endothelial specific knockdown of SOX17 show either no pulmonary hypertension or trends toward increases in basal RV systolic pressure and RV hypertrophy compared to wild-type mice but more severe pulmonary hypertension or earlier development of pulmonary hypertension in response to chronic hypoxia or sugen/hypoxia (Su/Hx-PH), suggesting that loss of endothelial SOX17 expression exacerbates pulmonary hypertensive responses (41, 43, 44). In contrast to other animal models of PAH, the pulmonary remodeling and right ventricular hypertrophy observed in these mice does not reverse following return to normoxia (41). *Sox17* deficient mice also have increased pulmonary inflammation as exemplified by increased perivascular infiltration of CD11b + cells (41). Pulmonary endothelial cells from *Sox17* knockout mice also demonstrate marked hyperproliferation and upregulation of inflammatory gene expression (42). In line with these results, autopsies from 4 out of 15 patients with PAH demonstrated marked decreases in *SOX17* expression in pulmonary arterial endothelial cell (PAEC) (41). Recent studies have also shown decreased *SOX17* gene expression and protein levels in pulmonary vascular endothelial cells (PVEC) isolated from PAH patients compared to failed donor lungs (34). Similarly, *SOX17* silencing mutations in human PAECs result in increased apoptosis, proliferation and adhesion (41). Conversely, Sox17 overexpression in mice attenuates hypoxia-induced PH and suppresses vascular remodeling, proliferation and inflammation in Sugen-hypoxia induced PH in an autocrine manner (44).

**Table 1:** Animals models of PAH with Sox17 inhibition.

Animal model	Cell type/knockout condition	Histologic/morphologic changes observed	Signaling pathways affected	Reference
– Adult Mice exposed to hypoxia	Sox17 deletion in pulmonary endothelial cells	Hypermuscularization, endothelial cell hyperplasia/proliferation, lung arteriolar inflammation, elevated RAP and RVH (by RVSP and RV/LV + septum ratio)	– Upregulation of HGF in Sox17 deficient EC after hypoxia – Inhibition of c-Met/HGF signaling reverses and prevents PH	Park et al., Circulation Research (41)
– In-silico electromobility shift assays of human PAECs – Adult Mice exposed to hypoxia and Su5416/hypoxia	Common human PAH risk *Sox17* enhancer region knockout in both hPAECs and adult mice	– Increased apoptosis, proliferation and disturbance of barrier function in hPAECs – Severe PH (by RVSP and RV/LV + septum ratio) in hypoxia and Su/hypoxia *Sox17ko* mice along with increased vessel muscularization and permeability	– Noted decreased binding of ROR*α* and HOXA5 TFs with Sox17 enhancer mutations	Walters et al., Circulation (42)
– Adult mice/rat exposed to hypoxia, MCT or 16α-hydroxyestrone (16 αOHE-estrogen metabolite)	– Conditional *Sox17* knockout or conditional transgenic overexpression	– Increased muscularization in pulmonary arterioles, elevated PA pressures /thickness/RVH and PH in *Sox17* knockout mice/rats exposed to hypoxia (by RVSP and RV/LV + septum ratio)	– HIF2α concentrations increased in lungs of *Sox17* knockout mice – Increased Sox17 expression promoted oxidative phosphorylation and mitochondrial function in PAECs – Male rats had higher Sox17 expression than female counterparts – Sox17 expression decreases with treatment with 16 αOHE and is increased in estrogen receptor knockout rats	Sangam et al., AJRCCM (43)
– Human endothelial cells from IPAH patients – Adult mice exposed to hypoxia	– Endothelial specific temporally controlled *Sox17* knockdown in mice – Knockout of *Sox17* in embryonic stages	– Increased spontaneous pulmonary artery muscularization and PH (by RVSP and RV/LV + septum ratio) in both embryonic and adult *Sox17* knockout mice and exaggerated hypoxia-induced PH in adult *Sox17* knockout mice (by RVSP and RV/LV + septum ratio) – Marked proliferation of endothelial cells with Sox17 deficiency both in HPVEC and mouse knockouts	– Increased paracrine mediated PASMC proliferation in-vitro in *Sox17* knockouts – Increased EC dysfunction in HPVEC that is mediated through E2F1 transcription factor (in-vitro dysfunction/proliferation and *Sox17* knockout PH rescued with E2F1 inhibition)	Yi et al., bioRxiv (34)
– Adult mice exposed to Su/Hypoxia	– Endothelial cell-specific knockdown or overexpression of *Sox17* via adenoviral vector siRNA	– Increased muscularization, RVH and RVSP in *Sox17* knockdown mice (by RVSP and RV/LV + septum ratio)	– SOX17 overexpression in HPAECs blocked VEGF-induced proliferation, serum free apoptosis and hypoxia/TNFalpha induced inflammation in a autocrine fashion – Exosomes from SOX17 overexpression in HPAECs prevented Su/hypoxia PH and reduced endothelial proliferation/inflammation – Protective affects appear to be in part mediated through micro RNA inhibition of NR4A3 and PCSK9	Zou et al., (44)
– HPAECs and HPASMCs – BMPR2 mutant mice	– Pulmonary artery on-a-chip model with HPAECs and HPASMCs separated by a membrane with ongoing pulsatile flow – Treated HPAECs with adenoviral mediated BMPR2 silencing RNA and hypoxia		– Decreased endothelial Sox17 expression observed in BMPR2 deficient HPAECs and endothelial cells of BMPR2 knockout mice. Associated with increased PASMC proliferation – Sox17 overexpression rescued PASMC proliferation and was associated with a significant increase in Prostacyclin synthase expression	Ainscough et al., (45)

**Figure 7:**
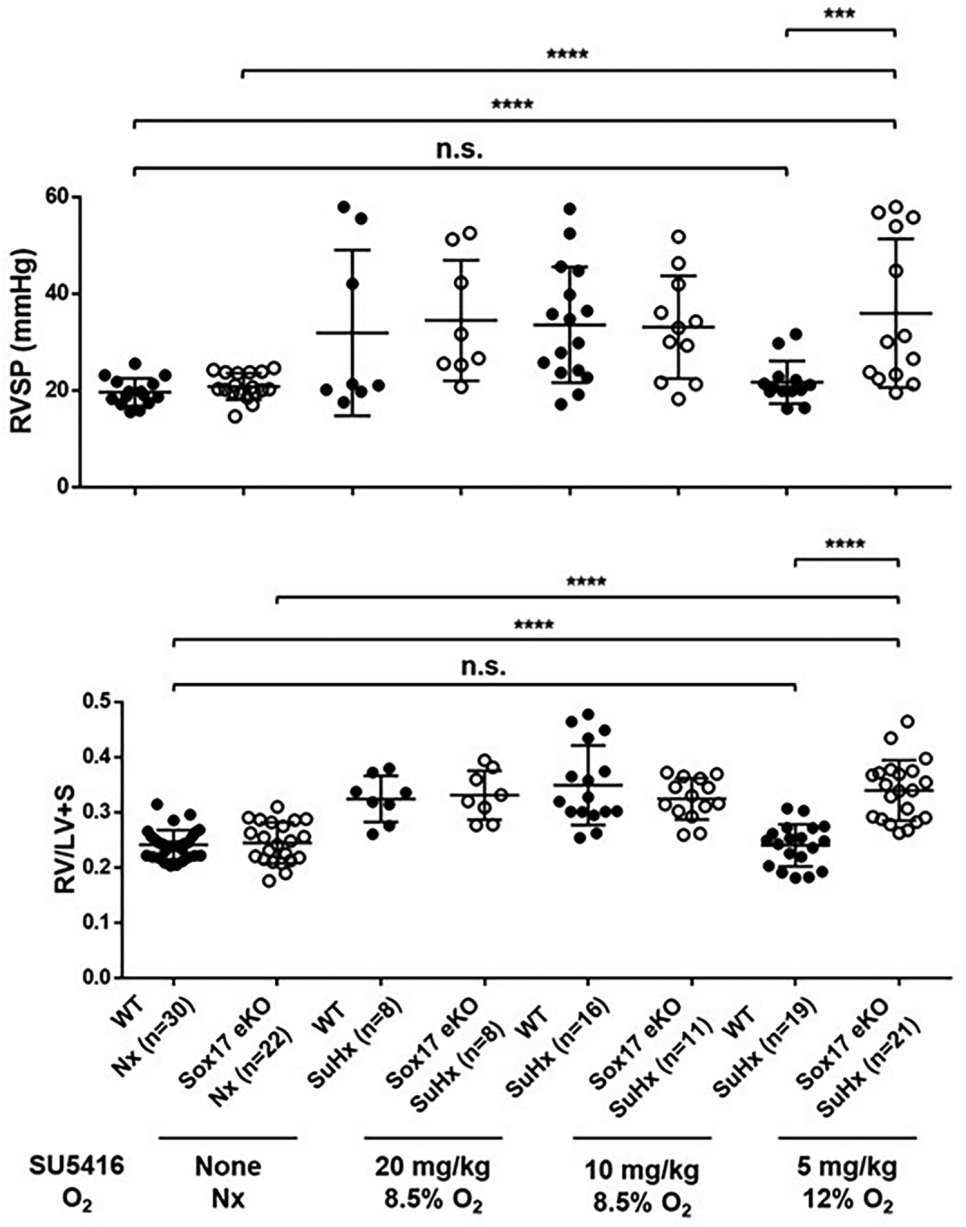
Exposure of SOX17 enhancer knockout mice model (eKO) to different levels of Sugen-5416 and hypoxia. (**A**) Right ventricular hypertrophy index (RV/LV + S). (**B**) Right ventricular systolic pressure (RVSP mmHg). SuHx, Sugen-5416 Hypoxia. O2, oxygen (Nx = normoxia). Numbers are shown on graphs. Ordinary One-way ANOVA. WT, wild type. KO, knockout. Reproduced from Walters et al., 2023.

In addition to animal models using conditional deletion of endothelial SOX17, transgenic mice engineered to resemble SOX17 mutations associated with increased risk of PAH are also more susceptible to pulmonary hypertension. *Sox17* enhancer knockout mice (*Sox17eKO*) generated by CRISPR-mediated knockdown of the *SOX17* enhancer regions initially detected in PAH patients (rs10958403 and rs765727) reduces Sox17 expression in mouse lung tissue and increases human pulmonary artery endothelial cell (PAEC) permeability while decreasing endothelial cell adhesion and VEGF-induced proliferative capacity (42). *Sox17eKO* mice develop normally but exhibit greater right ventricular hypertrophy and higher elevation of right ventricular systolic pressure (RVSP) compared to controls when exposed to hypoxia (44). They also demonstrate increased susceptibility to Sugen/hypoxia-pulmonary hypertension (42). In that study, low dose Sugen 5,416 (5 mg/kg) and mild hypoxia (12% oxygen) had no effect on wild-type mice but induced severe PH in *Sox17eKO* mice (Figure 7). These findings suggest that even when SOX17 is expressed in endothelial cells, mutations in the enhancer region that may impair normal activation of the SOX17 gene can increase the risk or severity of pulmonary hypertension.

SOX17 has been shown to be vital in numerous molecular signaling pathways important to PAH pathogenesis (Table 1, column 4). Important SOX17 downstream targets such as Notch signaling, BMPR2, estrogen receptor signaling, prostacyclin synthase and c-Met/HGF have been implicated in the endothelial dysfunction underlying Sox17's involvement in PAH.

SOX17 deficiency appears to upregulate endothelial cell hyperplasia and proliferation through upregulation of growth factor signaling in response to hypoxia or cellular stress. Interestingly, increased pulmonary vascular endothelial cell proliferation is observed in Sox17 deficient mice under hypoxic conditions (34). This appears to be due in part due to upregulation of Hepatocyte growth factor (HGF)/c-Met signaling. Along these lines, PAH can be reversed by suppression of c-Met via a small molecule inhibitor, crizotinib, in Sox17 knockout/hypoxic mice (41). Similarly, *SOX17* endothelial specific knockdown and deletion in both human and mice pulmonary vascular endothelial cells results in increased endothelial cell proliferation and smooth muscle cell proliferation. This endothelial cell dysfunction appears to act through E2F1, as its expression is upregulated in response to *SOX17* knockout and siRNA/small molecule inhibitor induced E2F1 knockdown rescues both endothelial dysfunction and *SOX17* deficient hypoxia-induced PH (34). This knockdown of SOX17 expression reduces BMPR2 expression and BMP9-induced phosphorylation of Smad1/5/9. Reciprocal results were also observed in BMPR2 deficient HPAECs, as decreased endothelial expression of SOX17 was observed in these cells and was associated with a paracrine mediated increase in pulmonary artery smooth muscle cell (PASMC) proliferation (45). Interestingly, in these experiments, overexpression of SOX17 in HPAECs rescued HPASMC proliferation and was associated with a significant increase in prostacyclin synthase. Similarly, hypoxia-inducible factors are transcription factors that upregulate angiogenesis, apoptosis and glycolysis in response to hypoxic stimuli. In *Sox17* knockout mice, HIF2alpha concentrations are increased in response to hypoxia and overexpression of Sox17 upregulates oxidative phosphorylation and mitochondrial function in endothelial cells (43). Interestingly, male rats appear to have increased Sox17 expression compared to their female counterparts, a finding which in part, may be explained by decreased Sox17 expression in the presence of estrogen metabolites. It is unlikely that sex related differences in SOX17 explain the higher incidence of PAH in woman compared to men, but raises an intriguing potential mechanism that may contribute to this well-known observation. Other potentially important roles for Sox17 include its ability to bind VE-cadherin and endothelial cell-selective adhesion molecules (ESAM) which are required to maintain blood vessel wall stability and permeability in the lung (46). Missense mutations in the highly conserved DNA-binding domain region of *SOX17* HMG box —similar to those described in humans, have been shown to impair direct DNA-binding and Beta-catenin protein complex interactions vital to its Beta-catenin, TGF-β and Notch signaling (47). Similarly SOX17 has been shown to be involved in other cellular pathways important to PAH pathogenesis such as cyclin, VEGF, Wnt/B-catenin, and endothelin signaling in the context of embryonic and arterial development. Evidence for Sox17's direct influence on these pathways in the context of PAH remains however limited or unexplored.

SOX17 expression is maintained in a restricted fashion in the adult endothelium, but increases in response to inflammatory or hypoxic signaling and deficiencies in expression appear to increase the likelihood of endothelial dysfunction and subsequent PAH. Given that relative deficiencies in SOX17 expression may predispose patients to PAH, increasing SOX17 expression or manipulating its downstream targets may be a novel approach to treating PAH. In-vitro and animal models have given rise to promising potential future therapies to rescue loss of SOX17 function in PAH. For example, in hPAECs with enhancer knockouts characteristic of PAH patients, Sirolimus and YK4279 [identified initially through high-throughput omics signatures using connectivity map (CMap)] were found to reverse *Sox17* enhancer knockout mediated repression (42). Similarly, inhibition of E2F1, a downstream target of Sox17, with a small molecular inhibitor (HLM) rescues PH in Sox17 deficient mice, reducing RSVP, pulmonary artery muscularization and pulmonary wall thickness (34).

Collectively, these results point to impaired SOX17 signaling as an important contributor to the pathogenesis of PAH. The major roles of SOX17 in vascular homeostasis, neovascularization and maintenance of arterial/endothelial identity as well as its importance in response to vascular injury and stress, make it a promising therapeutic target for PAH. At the same time, SOX17 knockout models may serve as important new preclinical models for investigating PAH.

## RUNX1

**Figure 8:**
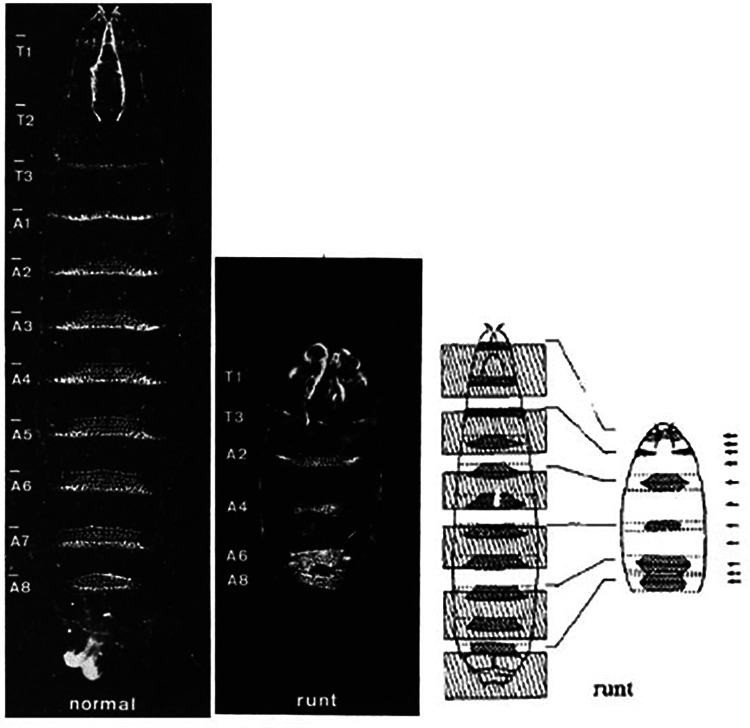
Representative images from Nusslein-Volhard's experiments on drosophila larvae. Normal larvae (left), Runx mutant larvae (middle) and authors representative depiction of inverted segment polarity and short segments (right). Reproduced from Nüsslein-Volhard et al., 1980.

Runt-related transcription factor 1 (RUNX1) is a downstream target of SOX17 and may play a role in mediating the effect of impaired Sox17 expression on PAH. The Runt gene was discovered in 1980 by Nusslein-Volhard and Wieschaus in a screen conducted to identify mutations affecting Drosophila larvae segment number, and polarity (48). One mutation resulted in pre-segmentation patterning defects that resulted in runted embryos—thus the Runt name was given to the mutated gene (Figure 8). Subsequent cloning revealed that the protein it encoded for was a transcription factor. RUNX1 was first identified in 1991 as a gene involved in the chromosome rearrangement t (8;21) associated with acute myeloid leukemia (49). Runx1 belongs to a transcription factor family called core-binding factors (CBF), which includes sequence-specific DNA-binding proteins Runx1-3. All Runx proteins share a non-DNA binding protein CBF-beta that helps them effectively bind DNA. RUNX1 is encoded by 12 exons and has various isoforms that can be synthesized by alternative splicing. Among these exons are two well defined domains: the runt homology domain (RHD) and the transactivation domain that bind DNA and facilitate protein-protein interactions, respectively. Runx1 partners with CFB-beta subunit to form a transcriptionally active heterodimer that can either activate or repress gene expression (50).

RUNX1 regulates the differentiation of HSCs into mature blood cells and is indispensable for the establishment of definitive hematopoiesis in vertebrates. RUNX1 is expressed in cells from all hematopoietic sites that contribute to formation of HSCs during development. HSCs are generated via a specialized subset of vascular endothelial cells known as the hemogenic endothelium that can differentiate into hematopoietic cells in a process called endothelial to hematopoietic transition (EHT). Developmentally, the endothelium arises in close contact with the hematopoietic system, and they share a common lineage in hemogenic endothelium. The adult hematopoietic system and long-term hematopoietic cells that give rise to life-long blood cell production are derived from hemogenic endothelial cells (HECs) in mid-gestational embryos. HECs are generated from embryonic endothelial cells after upregulation of Runx1 and activation of the Notch and TGF-β pathways during a brief developmental window in embryogenesis (Embryonic days 9.5–10.5). Runx1 is essential for EHT and its expression distinguishes HECs from non-HECs (51). HECs are located within the ventral aspect of the dorsal aorta and other large vessels. During EHT, HECs lose their tight junctions and demonstrate increased migratory behavior, characteristic of HSPCs. The HSPCs generated during EHT then seed the fetal liver and later the bone marrow (BM) to sustain hematopoiesis. EHT is a highly conserved stepwise process regulated by a complex interplay of a specific set of transcription factors, including Runx1, GATA1, GATA2, Lmo2, Scl/Tal1.

Homozygous RUNX1 mice lack hematopoiesis and are unable to survive past an early embryonic stage (E11.5-E12.5) (52) due to CNS hemorrhage. Heterozygous germline mutations in *RUNX1* are associated with Familial Platelet Disorder, a mild bleeding disorder associated with thrombocytopenia and high rates of myeloid leukemia (53). Other mutations in *RUNX1* are also associated with acute myeloid leukemia, T-cell acute lymphoblastic leukemia (present in ∼15% of cases), pancreatic cancer, myelodysplastic disorders, and a variety of other tumors (54).

Although EHT has been considered a developmental process that becomes inactive in adult life, under pathogenic conditions endothelial progenitor cell reservoirs may reactivate and contribute to adult hematopoiesis (55, 56). Furthermore, recent single-cell transcriptome expression analysis suggests that definitive hematopoiesis represents a continuum of phenotypes from endothelial cells to fully determined HSCs that undergo a continuous gain of specific lineage characteristics (57, 58).

Although initially thought to only be expressed in areas of hematopoiesis, in 2011 Heley et al., found that RUNX1 is also highly expressed in the developing human lung (59). Future work also uncovered that Runx1 is expressed in postnatal and adult murine lung and is highly upregulated in response to inflammation (60). Runx1 was also found to upregulate NF-kB and LPS-induced increases in macrophage IL-1B and IL-6 expression (61). At the same time, increased expression of Runx1 leads to enhanced endothelial cell proliferation and migration (62, 63).

In the pulmonary vasculature, injury to the endothelium followed by upregulation of inflammatory signals and recruitment of macrophages, neutrophils and inflammatory cells are thought to be sentinel events in the development of pulmonary hypertension. In accordance with this, lung inflammation precedes pulmonary vascular remodeling in experimental models of PAH and failure to resolve progression of this inflammatory response is thought to underlie the development of PAH (64). Pulmonary vascular lesions in PAH patients and animal models are characterized by progressive degrees of inflammatory/myeloid infiltrates including mast cells, dendritic cells, macrophages, T cells, B cells and lymphocytes. These infiltrates have corresponding increases in inflammatory cytokine and chemokine secretion, including IL-1, IL-6, IL-8, CCL5 and TNF-α; all of which are correlated with poor clinical outcomes in PH (65). In response to inflammatory signals and cells, pulmonary vascular cells can change their phenotype and cell fate and contribute to pulmonary vascular remodeling. Thus, transcription factors that direct stem cell differentiation from an endothelial to a myeloid fate may play important roles in the pulmonary vascular remodeling found in PAH.

Although the role of RUNX1 in the pathophysiology of PAH is not yet known, one hypothesis is that disruptions in endothelial progenitor cell (EPC) fate from definitive endothelium to hematopoietic or myeloid fate may explain inadequate vascular repair and perivascular infiltrates in patients with PAH. Interestingly, increased myeloid derived cells and increased myeloid specific transcription factors are seen in the perivascular infiltrates of PAH patients and animal models of PAH (66). Activation of developmental pathways is key in tissue regeneration in response to tissue injury. During injury, generation or dedifferentiation of cells into EPCs appears to be crucial for neovascularization. Maintenance of a differentiated endothelial fate for these progenitor cells however requires constant signaling. Defects in the maintenance of this fate or a predisposition to development of EHT and a shift toward myeloid differentiation may play a significant role in the abnormal pulmonary vascular remodeling that occurs in PAH.

## Runx1 in PAH

**Figure 9:**
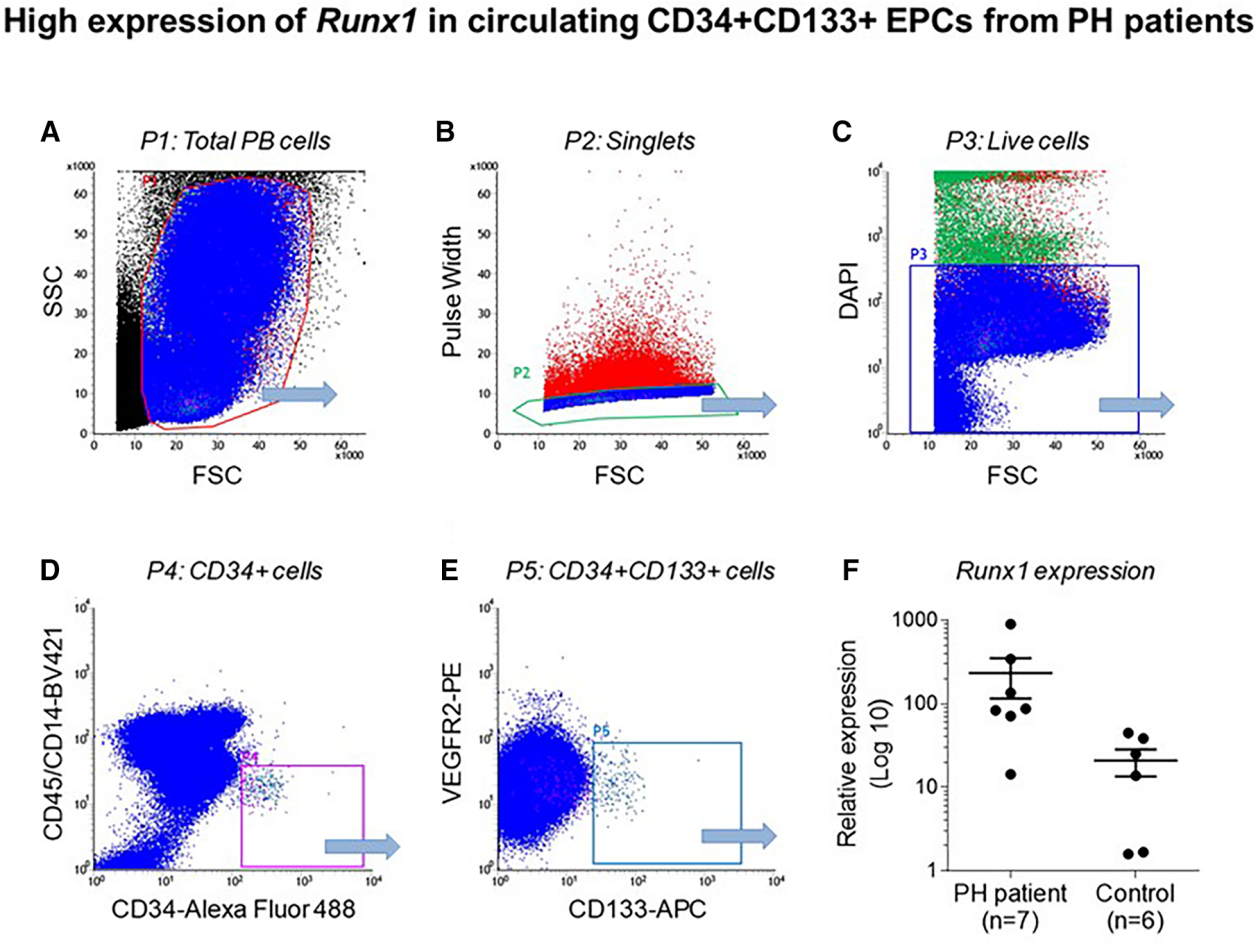
High expression of Runx1 in circulating CD34 + CD133 + EPCs from PH patients. Flow cytometry data showing (**A**) total peripheral blood (PB) cells (**B**) Single cells after doublet discrimination (**C**) Live cells (**D**) CD 34 + expression (**E**) CD34+, CD133+ and VEGF2R + cells and (**F**) Relative Runx1 expression in CD34+, CD133+, VEGF2R + cells in PH patients and controls. Reproduced from Liang et al., 2017.

**Figure 10:**
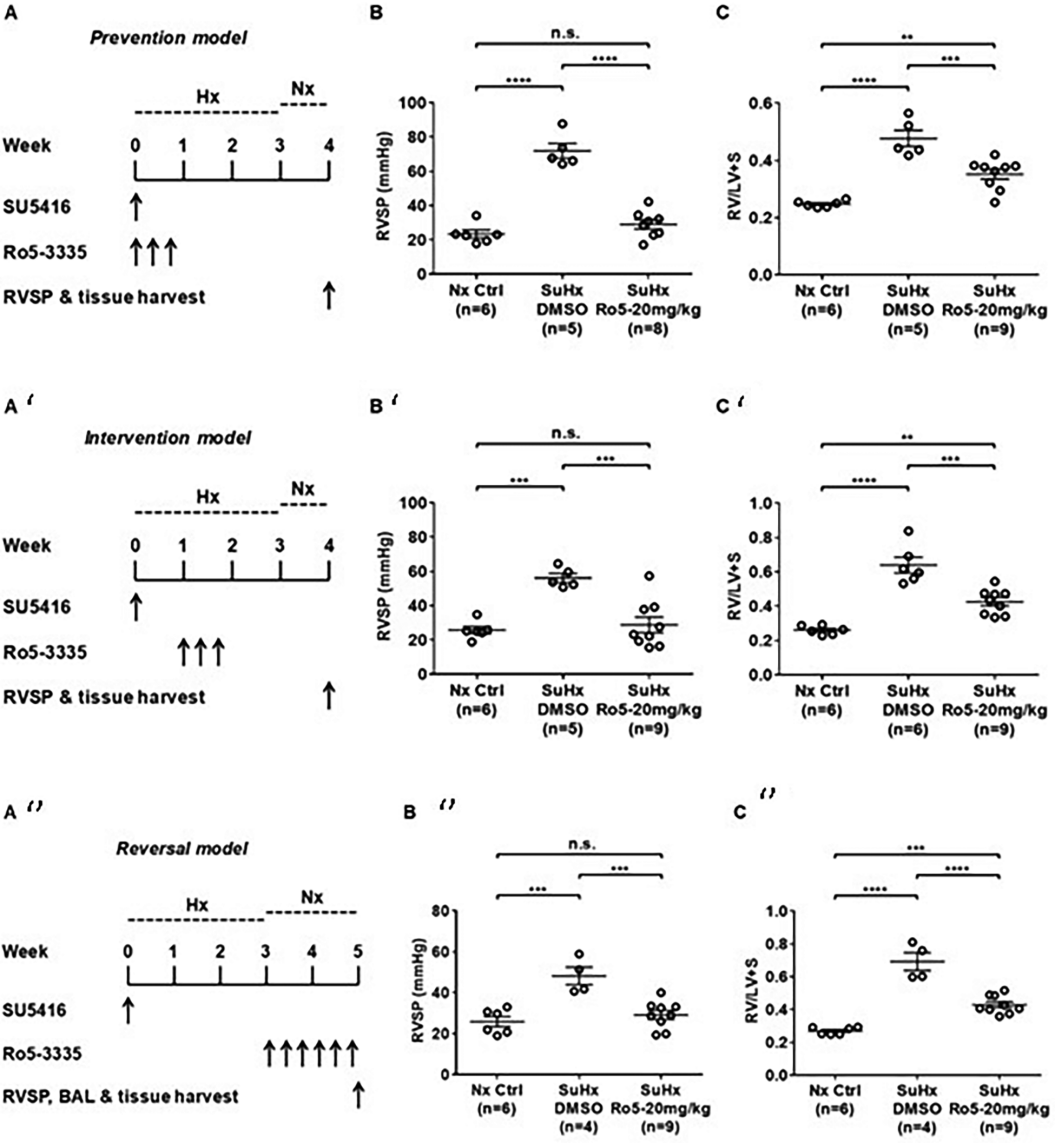
Runx1 inhibition prevents the development of and reverses established Su/Hx-PH. (**A**) Prevention model, SU5416 given along with Runx1 inhibitor (Ro5-3335) followed by subsequent measurement of (**B**) RVSP and C) RV/LV + S. (**A**’)Intervention model, SU5416 given followed by subsequent Runx1 inhibitor followed by subsequent measurement (**B**’) RVSP and (**C**’) RV/LV + S. (**A**’’)Reversal model SU5416 given followed by delivery of Runx1 inhibitor after development of PH followed by measurement of (**B**’’) RVSP and (**C**’’) RV/LV + S. RVSP, Right ventricular systolic pressure. RV/LV + S, Right ventricular hypertrophy index. BAL, bronchial alveolar lavage. SuHx, Sugen-5416. Hx, Hypoxia. Nx, normoxia. Reproduced from Jeong et al., 2022.

**Figure 11:**
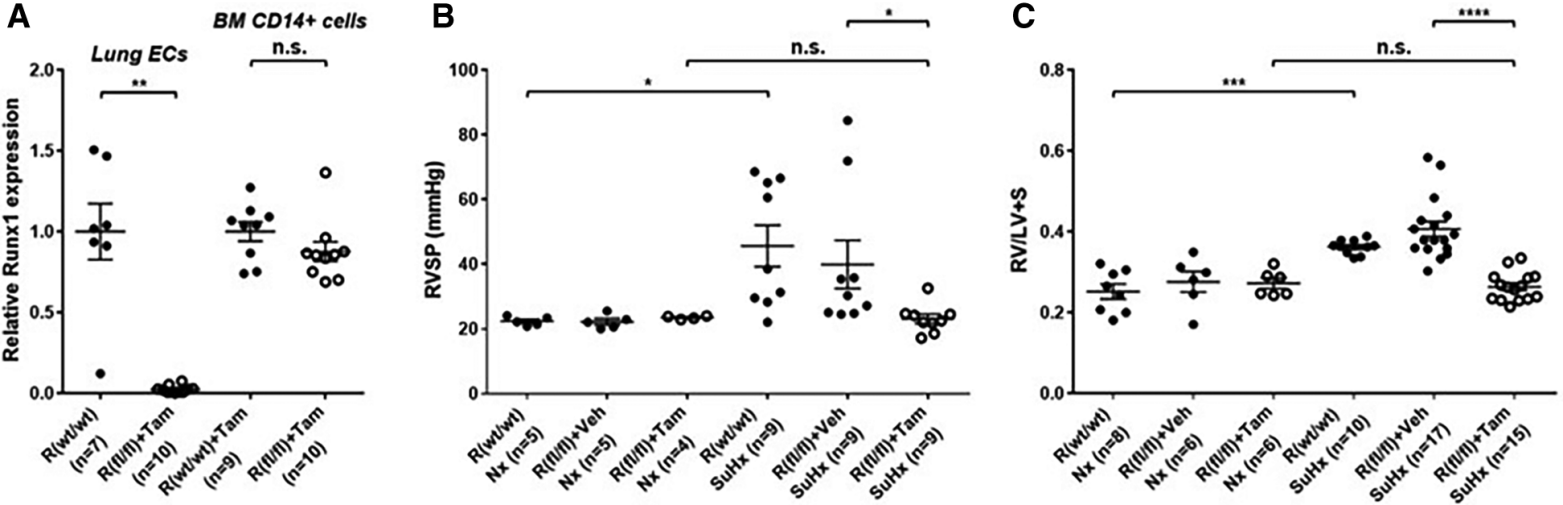
Genetic deletion of Runx1 in adult ECs/EPCs prevents SuHx-PH development in mice. (**A**) In 10- to 13-week-old mice, the loss of Runx1 in lung endothelial cells (ECs) upon Tam induction was verified per qRT–PCR. No changes in Runx1 gene expression in BM-derived CD14 + cells were found, demonstrating the endothelial specificity of Runx1 deletion in these mice. (**B,C**) Under Nx conditions, Cdh5-CreERT2; Runx1(wt/wt) mice, Cdh5-CreERT2; Runx1(fl/fl) mice treated with corn oil, and Cdh5-CreERT2; Runx1(fl/fl) mice treated with Tam all exhibited normal RVSP (**B**) and RV/LV + S ratio (**C**) Under SuHx conditions, Cdh5-CreERT2; Runx1(wt/wt) mice and Cdh5-CreERT2; Runx1(fl/fl) mice treated with corn oil exhibited significantly elevated RVSP (**B**) and RV/LV + S ratio (**C**) When the Cdh5-CreERT2; Runx1(fl/fl) mice were treated with Tam and placed under SuHx conditions, they exhibited normal RVSP (**B**) and RV/LV + S ratio (**C**) *P < 0.05, **P < 0.01, ***P < 0.001, ****P < 0.0001, n.s.: not significant, unpaired two-tailed Student's *t*-test (**A**) and ordinary one-way ANOVA with multiple comparisons (**B,C**), *n* = number of animals in each experimental group. Reproduced from Jeong et al., 2022.

Emerging data suggest that Runx1 plays a major pathogenic role in mouse models of PAH. Using conditional labeling of adult endothelial cells and lineage tracing (via VE-cadherin-cre; Zsgreen mice) in Su/Hx mice, we demonstrated that adult endothelial cells undergo hematopoietic transformation suggesting reactivation of EHT, and migrate from the BM to the pulmonary vasculature (55). We also found that RUNX1 expression is higher in circulating EPCs in mice with Sugen/hypoxia-induced pulmonary hypertension (SuHx-PH) and in patients with PAH, compared to EPCs obtained from control mice or healthy volunteers, respectively (Figure 9) (55). Other studies from our lab showed that Runx1 inhibition via small molecule inhibitor (Ro5-3335) blocks EHT *in vivo* and prevents both migration of EPCs from BM and the development of Su/Hx-PH- and monocrotaline-induced PH (MCT-PH) in mice (Figure 10) (55, 56). The prominent role of Runx1 in the pathogenesis of PH was further supported by recent studies in our lab that utilized lineage tracing of adult endothelium and conditional cell-specific Runx1 knockout mice. We found that targeted deletion of Runx1 in either myeloid or endothelial cells prevents the development of SuHx-PH (Figure 11). Interestingly, inhibition of Runx1 via Ro5-3335 also appeared to reduce perivascular macrophage recruitment and reverse established Su/Hx-PH in mice while dampening macrophage activation *in vitro* (55).

Collectively, these results indicate that Runx1 may play a crucial pathologic role in the development and progression of PH. Considering that Runx1 is a downstream target of Sox17, it is tempting to speculate that mutations in the Sox17 enhancer that are associated with increased risk of PAH, may predispose to PH by failing to suppress Runx1 expression. Studies in our lab aimed at testing this hypothesis by examining the effect of suppressed Sox17 expression on the expression of Runx1 in the lung and in progenitor cells of animals with pulmonary hypertension and patients with PAH are ongoing. If the findings further studies support this hypothesis, Runx1 inhibition may prove an effective approach to treating PAH.

## Bone marrow-derived progenitor cells

Although data from our lab suggests that Sox17 and Runx1 play an important role in the pathogenesis of PAH, the mechanism by which they do so is not clear. One possibility may be their role in driving HSCs toward either an endothelial or myeloid fate. The healthy endothelial monolayer of pulmonary arteries regulates the influx of fluid, proteins, and blood cells into surrounding parenchyma and maintains vascular tone and integrity. The gradual disruption of the endothelial layer in response to stress is thought to be one of the precipitating events in PAH. Loss of endothelial integrity eventually leads to the accumulation of pro-inflammatory cells, altered cell viability, smooth muscle cell hyperplasia, fibroblast proliferation and eventually occlusive vascular lesions, and increased vascular tone. At the same time, BM-derived myeloid cells play a prominent role in the perivascular inflammation that leads to remodeling of the extracellular matrix and adventitia. Emerging data suggest that local and BM-derived progenitor cells play important roles in homeostasis of the pulmonary endothelium and circulation that may be particularly important after injury that leads to PAH. Interestingly, SOX17 and RUNX1 are closely linked in EHT, as SOX17 has been shown to directly repress RUNX1 expression and induce human embryonic stem cells towards a hemogenic fate rather than an endothelial one (33). The ability of the Sox17/Runx1 axis to drive EPCs toward an endothelial or myeloid fate, may be one mechanism to explain how disrupted Sox17/Runx1 signaling results in pulmonary vascular remodeling.

The discovery of EPCs in peripheral blood during the late 1990s introduced the idea that vasculogenesis and angiogenesis did not occur only during fetal development but could arise in adulthood (67). The intense research into the origin and function of these cells opened a new era in theoretical regeneration of the cardiovascular (CV) system. However, confusion regarding the origin and identity of these cells quickly arose due to significant technological differences in how these cells were isolated, characterized and sustained in culture. Even today, there is no specific marker that clearly distinguishes a unique population of cells that can consistently be identified as EPCs. Nonetheless, their role in cardiovascular disease continues to be an active area of research and their role in the pathogenesis of PAH continues to be explored.

In 1997, Asahara et al. (68) characterized a population of progenitor cells expressing the cell surface markers CD34 and VEGFR2 which mobilized from the BM to the endothelium after ischemic injury and were capable of differentiating into endothelial cells that appeared to participate in vessel repair. These cells were termed EPCs. Traditionally, it was thought that injured endothelial cells in the pulmonary arteries were replaced by resident progenitor cells. However, since Asahara et al.'s findings, mounting evidence has indicated that circulating BM-derived EPCs have a prominent and pathologic role in PAH (69–71).

At the same time, our understanding of EPCs has changed significantly. Importantly, identification of EPCs in peripheral circulation via monoclonal antibodies and flow cytometry has been challenging as labeling of EPCs via CD34 positivity with endothelial antigens (i.e., VEGFR2, CD146, CD144, CD31, Tie-1, Tie-2) has shown significant overlap with markers on primitive HSCs. Furthermore, once isolated, these cells behave like hematopoietic colony forming cells and do not form endothelial cells (72). An alternative approach to isolating EPCs is based on isolation of circulating peripheral blood mononuclear cells (PBMCs) and growing them in pro-angiogenic cell culture media. Interestingly, EPCs obtained through this method comprise two relatively discrete populations. First described by Kalka et al. as “early” and “late” EPCs based on the time of the appearance of endothelial cells *in vitro* (73). Early EPCs are characterized by detection of endothelial cells after 7–10 days of growth and display limited endothelial proliferative capacity but have strong pro-angiogenic paracrine activity. The majority of these early EPCs display markers similar to those of monocyte/macrophage and lymphocyte lineage. Conversely, late EPCs develop endothelial cell characteristics after three weeks of growth and display high endothelial proliferative potential and express endothelial cell lineage markers.

The means by which EPCs influence PAH remains unclear. Current data support 4 main mechanisms: (1) localization to areas of vascular injury and restoration of vascular integrity; (2) paracrine effects—potentially mediated by secretions of proangiogenic growth factors; (3) support/restoration of other cells, and (4) differentiation into proinflammatory cells with subsequent immunomodulatory effects.

Early studies on the vascular reparative effects of EPCS were conducted in the systemic circulation. These studies demonstrated that BM derived EPCs were recruited to the site of vascular injury following wire disruption of the femoral artery in mice (74). In 2005, Hayashida et al. used green fluorescent protein (GFP)-labeled BM cells to demonstrate a similar recruitment of BM-derived progenitor cells to the pulmonary circulation during the development of PH. They showed a striking increase in BM-derived cells in the pulmonary arteries of mice after induction of hypoxic PH. GFP positive cells were recruited to the distal pulmonary circulation and appeared to be involved in pulmonary vascular remodeling (75). Similarly, in calves with hypoxic pulmonary hypertension, a marked increase in BM derived c-kit + stem cells was seen in peripheral blood with a corresponding decrease in c-kit + cells in BM (76). BM-derived EPCs have also been identified in the pulmonary vasculature in other pre-clinical models of PAH including MCT and Sugen/hypoxia (76, 77), as well as in patients with PAH (78). Recruitment of BM-derived progenitor cells to areas of vascular injury is driven in part by chemokine gradients. In particular, vascular injury is associated with increased expression of CXCL12, the ligand for the CXCR4 receptor expressed by HSC (79). Pulmonary vascular expression of both CXCL12 and CXCR4 has been demonstrated in lungs from rats with Sugen/hypoxia PH and in patients with PAH (80) and inhibition of the expression or binding of CXCR4/CXCL12 reverses MCT-induced PH in rats (81). Interestingly, EPCs from patients with PAH have increased proliferative capacity compared to control patients (82). Furthermore, the number of EPCs in PAH patients with BMPR2 mutations are increased and when grown *in vitro* demonstrate a hyperproliferative phenotype and an inability to form vessel networks (83).

**Figure 12:**
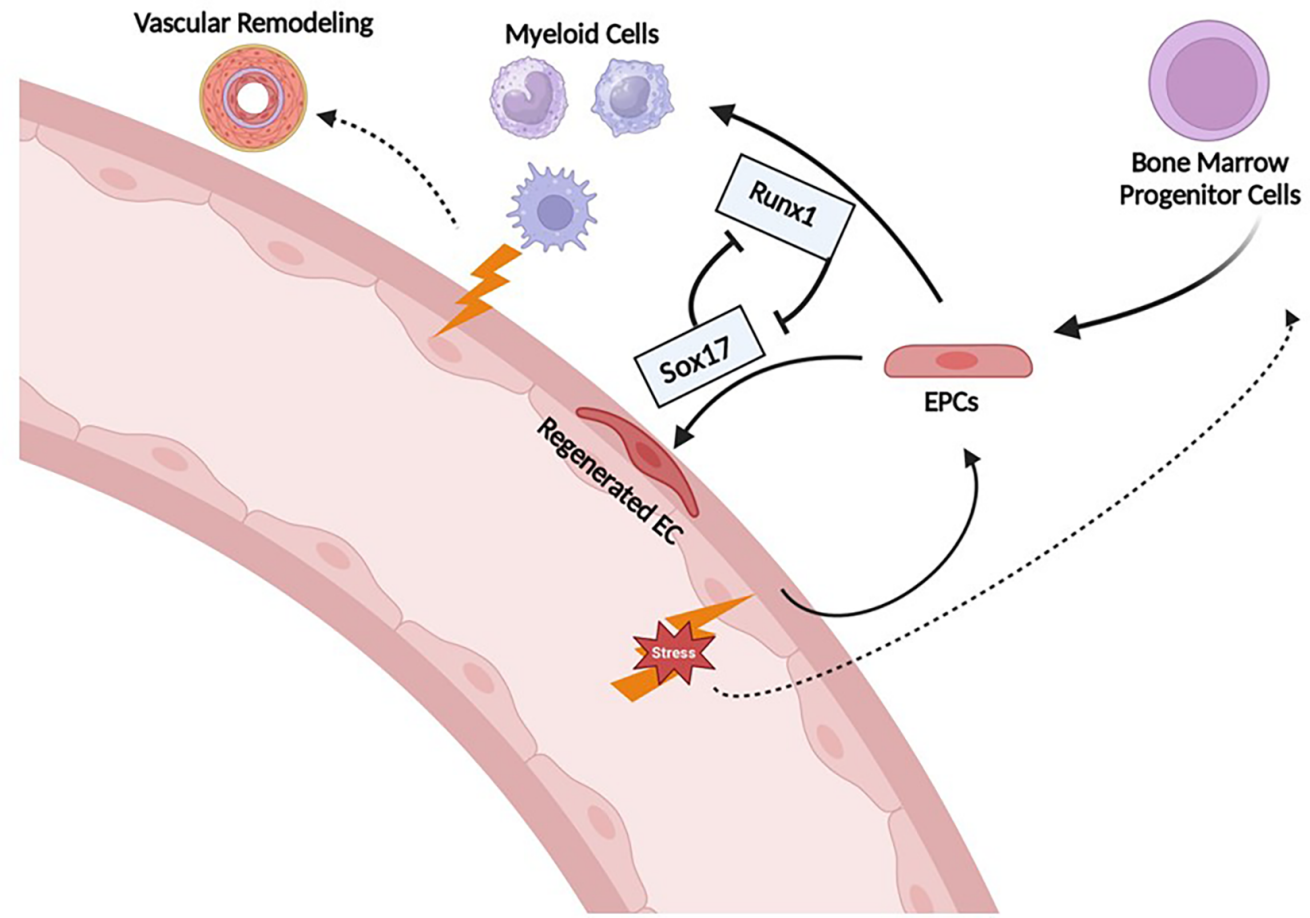
Proposed mechanism of Runx1 and Sox17 involvement in endothelial cell regeneration after injury. Sox17 is necessary for EC regeneration following injury. Runx1 skews differentiation towards HSC/myeloid lineage which may contribute to further inflammation/propagation of PAH. EPCs, endothelial progenitor cells; EC, endothelial cells.

A pathogenic role of BM-derived EPC has been suggested by a number of studies in which these cells have been shown to induce PH when transplanted into healthy animals. For example, our lab demonstrated that whole BM obtained from mice with MCT-PH induces PH when transplanted into healthy mice (84, 85). Transplanting BM from control mice into healthy mice had no effect. Similarly, BM from pulmonary hypertensive BMPR2 heterozygote mice induce PH in a dose-dependent manner and increase the number of donor-derived inflammatory cells in the pulmonary vasculature when transplanted into healthy control mice (81). In contrast, transplantation of BM from wild-type mice into BMPR2 heterozygotes attenuates the severity of PH. Whether the latter result is due to a healing effect of the BM cells from wild-type mice or the destruction of pathogenic EPC in the PH mice is unclear, but these transplantation experiments require non-lethal radiation prior to transplant which destroys most of the native BM cells. Our group has also demonstrated that non-lethal irradiation alone (i.e without subsequent BM transplant) can partially reverse SuHx-PH (86). Whether or not it is the EPC fraction that was responsible for BM inducing PH in the above experiments is uncertain. However, we demonstrated that depletion of EPC, defined as ckit+/sca1+/vegfr2+, from the BM of MCT-PH mice prior to transplantation prevented induction of PH in the healthy mice (85). Finally, EPCs defined as CD133 + obtained from the BM of PAH patients cause pulmonary vascular endothelial injury, *in situ* thrombi, right ventricular hypertrophy and increased mortality when xenografted into immunodeficient mice, whereas EPCs obtained from patients without PAH do not. Interestingly, these CD133 + cells expressed increased myeloid-specific transcription factors (GATA1, EKLF, Fli) compared to controls (85, 87). Collectively, these results demonstrate that EPCs obtained from animals with PH and patients with PAH are capable of inducing PH, and causing pulmonary vascular remodeling. However, the mechanisms responsible for driving EPCs from a reparative response toward pathologic remodeling is not clear. Previous studies have shown that SOX17 plays a key role in the induction and expansion of arterial endothelial precursors derived from CD34^+^ progenitors in human umbilical cord blood or adult BM (88) and we have shown that Runx1 expression is higher in circulating EPCs obtained from PAH patients compared with controls. Together, these findings support the hypothesis that aberrant pulmonary vascular remodeling in PAH may be due in part to imbalances in SOX17 and RUNX1 expression in EPCs that leads to suppression of endothelial differentiation and instead drives them toward a hematopoietic fate (Figure 12).

## Future directions

Evidence is accruing to support a role for SOX17 and RUNX1 in the pathogenesis of PAH. Further research is needed to elucidate the mechanisms by which these developmentally important transcription factors may modulate pulmonary vascular remodeling and the development or progression of PAH. Whether or not the increased risk of PAH associated with mutations in the *SOX17* gene is due to insufficient repression of its downstream target *RUNX1* is not known. RUNX1 expression in the lung or circulating progenitor cells has not been examined in patients with PAH associated with SOX17 mutations, although this is an ongoing area of research in or lab and by other investigators. In addition to RUNX1, numerous other targets of SOX17 including HGF/c-Met, E2F1, BMPR2, and estrogen metabolism have been shown to play significant roles in modulating pulmonary hypertensive responses, and perturbations in their expression may play important roles in PAH associated with impaired SOX17 expression. Furthermore, if insufficient suppression of RUNX1 by impaired SOX17 expression causes pulmonary hypertension, it is not clear whether impaired SOX17 and RUNX1 signaling fails to maintain pulmonary vascular endothelial cell homeostasis or drives differentiation of BM-derived progenitor cells toward a myeloid fate. However, there is strong and accruing evidence to support the pathogenic role of SOX17 and RUNX1 in PAH and the potential of targeting the SOX17 and RUNX1 axis as a novel therapeutic approach in the management of PAH. Future studies are needed to determine if enhanced expression of SOX17 or inhibition of its downstream targets such as RUNX1, HGF/c-Met, or E2F1 can reverse PAH associated with the rare or common variants of *SOX17* mutations or in patients with PAH that are not associated with impaired SOX17 expression. To this end, RUNX1 inhibitors have been developed for the treatment of hematologic malignancies and may be repurposed for the treatment of PAH and recent studies have suggested several drugs that have potential to enhance SOX17 expression despite mutations in enhancer signal 1 and 2 (42). Targeting transcription factors that are intimately involved in endothelial generation and repair such as SOX17 and RUNX1 has the potential to not only shed new light on the pathogenesis of PAH but to significantly change the approach to treating this devastating disease.
